# Optical coherence tomography imaging device comparison study in papilloedema secondary to idiopathic intracranial hypertension

**DOI:** 10.1038/s41433-026-04823-4

**Published:** 2026-09-22

**Authors:** Gabriele Berman, Andreas Yiangou, Mark Thaller, Nick Capewell, Donna Holding, Georgios Tsermoulas, Alexandra J. Sinclair, Richard J. Blanch, Susan P. Mollan

**Affiliations:** 1 Birmingham Neuro-Ophthalmology, University Hospitals Birmingham NHS Foundation Trust, Queen Elizabeth Hospital, Birmingham, UK; 2 Metabolism and Systems Science, School of Medical Sciences, University of Birmingham, Birmingham, UK; 3 Department of Neurology, University Hospitals Birmingham NHS Foundation Trust, Birmingham, UK; 4 Department of Neurology, Sandwell and West Birmingham NHS Trust, Birmingham, UK; 5 Department of Neurosurgery, University Hospitals Birmingham NHS Foundation Trust, Birmingham, UK; 6 NIHR Birmingham Biomedical Research Centre, University Hospitals Birmingham NHS Foundation Trust and University of Birmingham, Birmingham, UK; 7 Neuroscience and Ophthalmology, School of Infection, Inflammation and Immunology, University of Birmingham, Birmingham, UK; 8 Academic Department of Military Surgery and Trauma, Royal Centre for Defence Medicine, Birmingham, UK; 9 Ophthalmology Department, Queen’s University, Kingston, ON Canada

**Keywords:** Optic nerve diseases, Medical imaging

## Abstract

**Background:**

Optic nerve imaging assessing papilloedema is an increasingly important clinical measure to guide treatment. The aim of this study was to understand the agreement between two commonly used OCT devices and whether pooling of papilloedema OCT imaging data could be easily facilitated.

**Methods:**

People living with idiopathic intracranial hypertension (IIH) underwent non-dilated same-day OCT imaging performed on Spectralis SD-OCT (Heidelberg Engineering, Heidelberg, Germany) and Cirrus 6000 OCT (Carl Zeiss Meditec, Dublin, CA). Comparable measurements were collected (retinal nerve fibre layer (RNFL), maximum height of the optic nerve head and macular ganglion cell layer assessment). Bland-Altman plots were constructed, intraclass correlation coefficient (ICC) and Pearson correlation coefficient were calculated.

**Results:**

Ninety-seven people with a definite diagnosis of IIH were included: 95% were women with a mean age of 31 years. Mean global RNFL thickness was 117.4μm (SD 41.6) on the Spectralis platform and 122.5μm (SD 53.8) on the Cirrus platform. There was excellent agreement between the devices for RNFL thickness measurements (ICC = 0.922), although the Bland-Altman plot revealed increasing discrepancy at higher RNFL thicknesses. Maximum disc height displayed good agreement (ICC = 0.882) between devices. There was a very strong positive correlation between Spectralis GCL volume and Cirrus GC-IPL thickness(r = 0.911).

**Conclusions:**

This OCT imaging dataset spanning a range of papilloedema severity in people with a definite diagnosis of IIH showed good relative agreement of RNFL measurements between Spectralis and Cirrus devices at RNFL thicknesses close to normal, but greater variance with more severe papilloedema. RNFL measurements from the two devices cannot be used interchangeably. Macular GCL assessments showed a strong positive linear correlation.

## Introduction

Optical Coherence Tomography (OCT) imaging is becoming increasingly important in clinical decision-making in neuro-ophthalmology [1, 2] and is essential in the management of idiopathic intracranial hypertension (IIH). Retinal nerve fiber layer (RNFL) thickness, total retinal thickness, and macular ganglion cell layer (GCL) volume predict longitudinal changes in Humphrey visual field mean deviation [3, 4]. Optic nerve head parameters may additionally serve as surrogate markers of intracranial pressure change [5]. Several different OCT imaging devices are commonly used in clinical practice; however, the published literature reports little recognition that the most cited measurements may not be interchangeable between platforms in neuro-ophthalmic disease, including papilloedema. To ensure optimal patient outcomes, the measurements that guide treatment need to be accurate, but also comparable to those reported in the published evidence that guides practice. There is therefore an unmet need to demonstrate that the devices used routinely in clinical practice and for research outcomes have high levels of agreement. Evidence evaluating optic nerve and retinal OCT measures across devices is sparse, with most studies conducted in people with multiple sclerosis [6–8].

Quantitative repeated structural measurements on the same scale should have absolute agreement [8]. Therefore, it would be expected that measures such as the RNFL and maximum height at the optic nerve head between devices should have (near) absolute agreement. However, different magnitudes of measurement for RNFL thickness have been demonstrated in the eyes of patients with glaucoma [9] and healthy controls [10]. In neuro-ophthalmic practice, the measures most commonly used to assess patients with papilloedema are RNFL thickness, maximum disc height, and macular ganglion cell layer assessments[11].

We aimed to explore the agreement between two commonly used spectral-domain OCT devices (Heidelberg Spectralis and Zeiss Cirrus 6000) for measurements of peripapillary RNFL thickness, maximal disc height and macular ganglion cell layer assessment in people with IIH and varying levels of optic nerve elevation.

## Methods

### Participants

Inclusion criteria: Historically consented patients aged >18 years meeting diagnostic criteria for IIH attending a neuro-ophthalmology outpatient clinic. All patients had a definite diagnosis of IIH by the revised diagnostic criteria for IIH that define the presence of papilloedema at diagnosis; neuroimaging that had excluded venous sinus thrombosis and pathological structural lesions; and an elevated lumbar puncture opening pressure (LP OP) ≥25 cmCSF in a properly performed LP with the cerebrospinal fluid (CSF) composition being normal[12, 13]. Data were collected prospectively from a registry database of people with IIH (IIH:Life). The IIH:Life study is an ongoing prospective observational cohort study of people with IIH attending a specialist clinic at a single tertiary neuroscience centre in the United Kingdom (University Hospitals Birmingham NHS Foundation Trust) [4, 14, 15].

### Data collected

Data were collected at the time of the participant’s routine clinical outpatient visit. Age, sex, LP OP at diagnosis and body mass index (BMI) were recorded. Disease duration was reported in months from time of diagnostic LP to the time of OCT imaging.

### OCT imaging

**Fig. 1:**
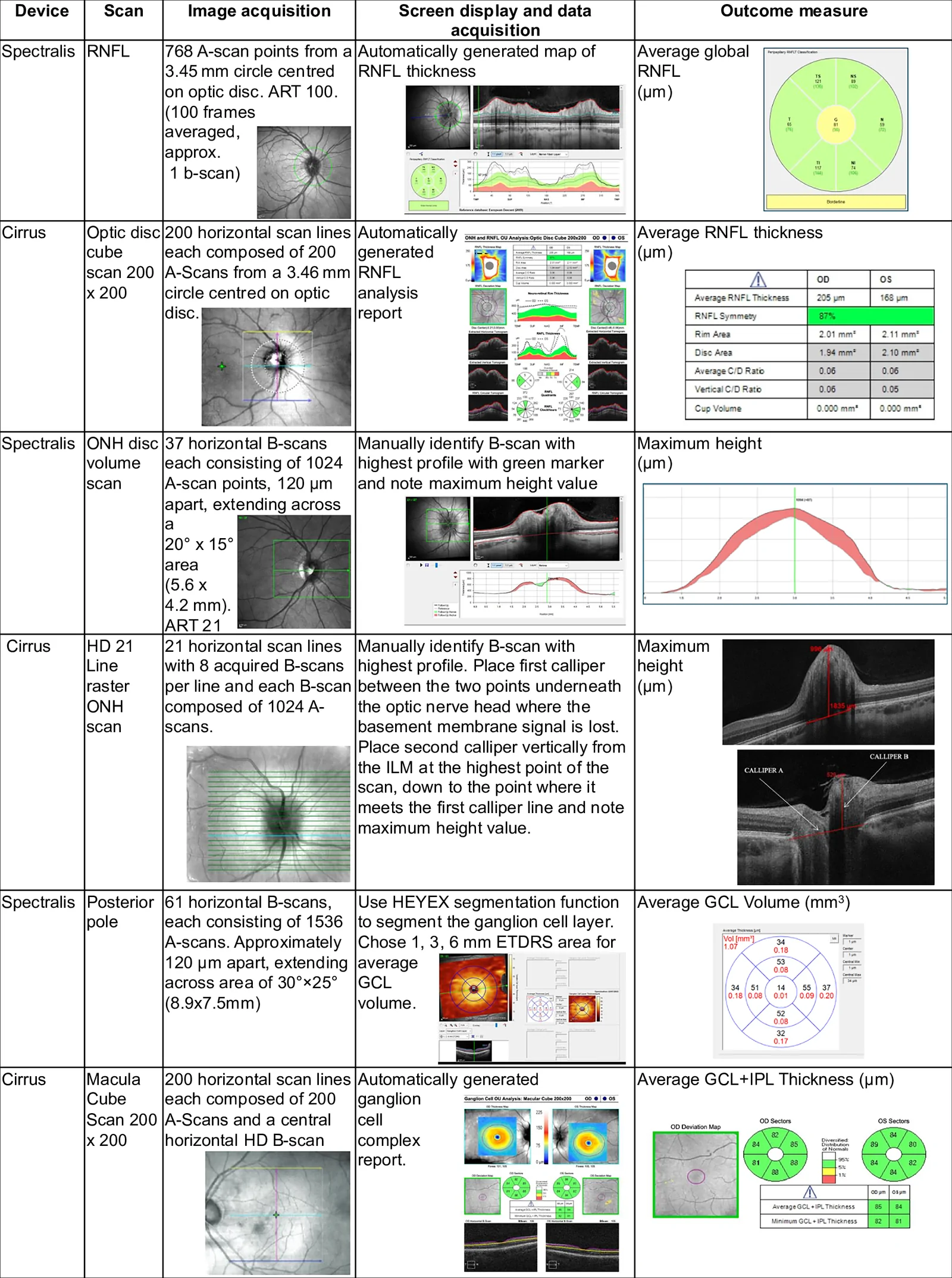
Overview of scanning protocol for Heidelberg Spectralis and Zeiss Cirrus 6000 SD-OCT. Abbreviations: ART automatic real time, ETDRS Early Treatment Diabetic Retinopathy Study, HD high definition, GCL ganglion cell layer, ILM internal limiting membrane, IPL internal plexiform layer, pRNFL peripapillary retinal nerve fibre layer, RNFL retinal nerve fibre layer, SD spectral domain, OCT optical coherence tomography, ONH optic nerve head.

Adults (age >18 years) with a definite diagnosis of IIH underwent non-dilated same day spectral-domain OCT imaging performed on Spectralis SD-OCT (Heidelberg Engineering, Heidelberg, Germany) and Cirrus SD-OCT 6000 (Carl Zeiss Meditec, Dublin, CA) devices at a single centre (Fig. 1). The Spectralis SD-OCT is a dual-beam OCT that utilises confocal laser-scanning ophthalmoscope with a wavelength of 870 nm and an infrared reference image to obtain images of ocular microstructures with an acquisition rate of 40,000 A-scans per second and an axial/transverse resolution of 7/14 µm (https://business-lounge.heidelbergengineering.com/us/en/products/spectralis/spectralis/#product-details). The Cirrus SD-OCT utilises a 20 Hz line scanning ophthalmoscope with a wavelength of 840 nm and an axial/transverse resolution of 5/15 µm (https://www.zeiss.com/meditec/en/products/optical-coherence-tomography-devices/cirrus-6000-performance-oct.html). All measurements were performed on the same day within minutes of each other. Comparable parameters measured were: global RNFL thickness, maximum disc height and GCL volume (Spectralis) and RNFL thickness, maximum disc height and GCL-IPL thickness (Cirrus). Reporting adhered to the Advised Protocol for OCT Study Terminology and Elements (APOSTEL) recommendations [16] (Supplementary table 1).

### Acquisition of Heidelberg Spectralis SD-OCT data

#### Retinal nerve fibre layer

To acquire the RNFL image with the Spectralis SD-OCT a scan circle with a diameter of 3.45 mm was manually positioned at the centre of the optic disc. Images at the same location were captured and averaged automatically by the built-in software to increase the image signal-to-noise ratio and improve the quality of subsequent images. All captured images had a signal quality ≥ 20 dB. Automated real-time averaging (ART) was 100. To determine the global RNFL thickness, the automatic segmentation was checked and if erroneous, it was manually adjusted by a masked observer to ensure accuracy before recording the global RNFL value (Fig. 1).

#### Maximum height

To determine the maximum height, the Heidelberg Eye Explorer Glaucoma module optic nerve head scan was used. This was set to a 5.6 ×4.2 mm rectangular frame over the optic disc, with 37 horizontal B-scans and ART mode of 21 images. To determine the maximum height, the thickness profile tab was used to review all B-scans (each B-scan was checked for accurate segmentation). The B-scan that subjectively appeared to have the thickest profile was selected. The green marker was dragged to the position where the disc appeared to be at its highest point, and a note was made of the measurement next to the marker. This was repeated for any other B-scans that had a similar subjective profile and the largest value found was recorded (Fig. 1).

#### Ganglion cell layer

The Heidelberg Spectralis Posterior Pole scan provided a 30° x 25° volumetric structural image of the retina, consisting of 61 B-scans and ART mode of 9 images. Using the HEYEX segmentation function, the GCL was isolated for analysis. The Early Treatment Diabetic Retinopathy Study grid (1-, 3-, 6-mm) was used to determine the total GCL volume (Fig. 1).

### Acquisition of Zeiss Cirrus SD-OCT data

#### Retinal nerve fibre layer

Unlike Spectralis, which allows explicit ART averaging of B-scans, Cirrus utilises inbuilt proprietary internal averaging and enhanced sampling to improve signal-to-noise and does not allow user-defined ART settings per scan. The optic disc cube (200×200 A-scans) protocol was used for image acquisition. The protocol generated a cube of data through a 6 mm square grid by acquiring a series of 200 horizontal scans. For analysis, the Cirrus algorithm identified the centre of the optic disc and automatically placed a calculation circle 3.46 mm in diameter around it. Scans with signal strength <6/10 were repeated for better quality. An automatically generated analysis report (ONH and RNFL Analysis) was exported, and the average RNFL thickness was recorded (Fig. 1).

#### Maximum height

A HD 21 Line raster ONH scan composed of 21 horizontal B-scans provided a structural volumetric image of the optic nerve head. To determine the maximum height, all B-scans were manually reviewed to identify the B-scan with the highest profile. Using two calipers, the first caliper was placed horizontally between the two points of Bruch’s membrane opening where the basement membrane signal was lost and the second caliper was placed vertically from the internal limiting membrane at the highest point of the scan, down to the point where it met the first caliper line to record the maximum height (Fig. 1).

#### Ganglion cell complex (GCL + IPL)

The Cirrus SD-OCT Macula Cube Scan 200 ×200 was used to capture a structural image of the retina composed of 200 horizontal scan lines with a central horizontal HD B-scan. From the automatically generated GCC report, the average GCL + IPL thickness was recorded (Fig. 1).

#### Statistics

The aim of the study was to evaluate levels of agreement between two instruments and imaging data from both eyes were therefore treated as independent images [17]. Mean values and SD were used for continuous variables. Intraclass correlation coefficient (ICC; two-way mixed, average measure, absolute agreement) [18] was used to address quantitative repeated measurements on the same scale (RNFL and maximal height, µm) and Pearson’s r correlation coefficient [19] was calculated for measurements on different scales (GCL volume, mm^3^ and GCL + IPL thickness, µm) and strength of association assessed according to prior convention [18–20]. Mean difference and 95% limit of agreement (LoA) were used to assess inter-device agreement. The 95% LoA was calculated as mean ± SD×1.96 of differences between paired measurements and illustrated in Bland-Altman plots. Statistical evaluation was performed in Microsoft Excel (version 16.105) and IBM SPSS Statistics for Windows (version 30.0) and a *P*  <  0.01 was defined as statistically significant.

## Results

A total of 97 people with a definite diagnosis of IIH were included, of whom 95% were women. The cohort had a mean age of 30.2 years (SD 8.6) with mean BMI of 39.2 kg/m^2^ (SD 8.0), mean LP OP 36.1 cm CSF (SD 7.1) and mean disease duration of 33 months (SD 39; Table 1).

**Table 1:** Baseline characteristics of 97 patients with idiopathic intracranial hypertension undergoing same-day OCT imaging.

	Mean (SD)	Number (N)
Age, years	30.2 (8.6)	*N* = 97
Women, n	92/97 = 94.8%	*N* = 97
BMI, kg/m^2^	39.2 (8.0)	*N* = 90
LP OP, cmCSF	36.1 (7.1)	*N* = 77*
Disease duration, months	32.8 (38.7)	*N* = 72*

*Note that there was missing data as the LP was performed at different hospital sites and reported to the clinic as either high or above 25 cmCSF without the actual LP OP measurement. Disease duration could not be accurately calculated if the date was not supplied to the clinic by either the patient or the medical notes.

*BMI* body mass index, *CSF* cerebrospinal fluid, *LP OP* lumbar puncture opening pressure, *SD* standard deviation.

Mean RNFL thickness was 117.4μm (SD 41.6) (Spectralis) and mean RNFL thickness was 122.5μm (SD 53.8) (Cirrus; Table 2). The mean maximum disc height was 803.7 ± 149.7μm on the Spectralis platform and 743.5 ± 155.2μm on the Cirrus platform. Mean GCL volume was 1.10 ± 0.11mm^3^ on Spectralis and mean GCL + IPL thickness was 81.5 ± 10.0μm on Cirrus. The mean difference in RNFL thickness was –4.96 ± 18.1μm thinner on Spectralis (95% CI –40.4–30.5 μm). The mean difference in maximum disc height was 57.4 ± 48.4μm thicker on Spectralis (95% CI –37.6–152.3 μm).

**Table 2:** Average (SD) same-day measurements (RNFL, maximum height and GCL volume and GC-IPL thickness) performed on Spectralis and Cirrus.

Device measurement	Spectralis	Cirrus	Device measurement
Global pRNFL, μm (SD)	117.4 (41.6)	122.5 (53.8)	Average RNFL thickness, μm (SD)
Maximal height, μm (SD)	803.7 (149.7)	743.5 (155.2)	Maximal height, μm (SD)
GCL volume, mm^3^ (SD)	1.10 (0.11)	81.5 (10.0)	GC-IPL thickness, μm (SD)

*GCL* ganglion cell layer, *GC-IPL* ganglion cell layer-inner plexiform layer, *RNFL* retinal nerve fibre layer, *SD* standard deviation.

**Fig. 2:**
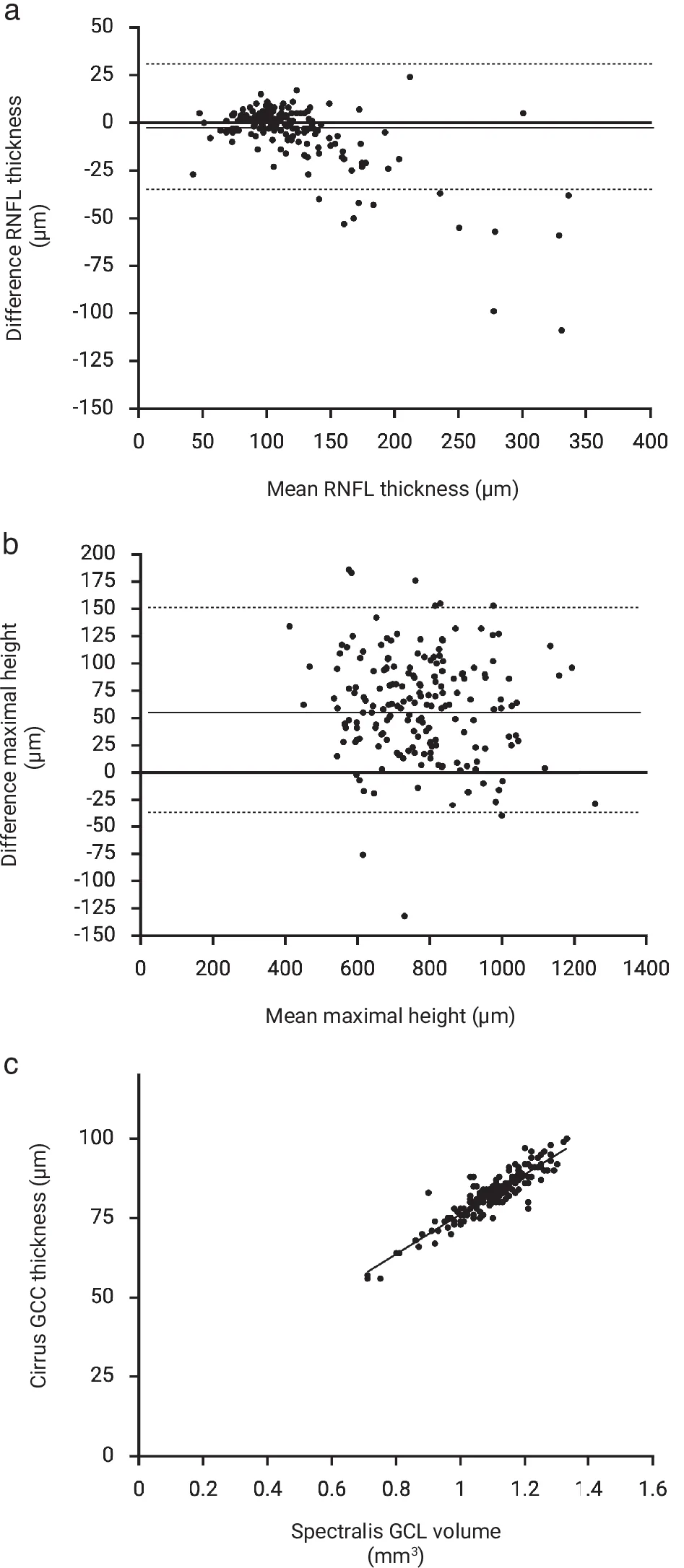
This is a Bland-Altman plot of the RNFL thickness agreement between the Spectralis and Cirrus. **A** Bland-Altman plot of the RNFL thickness agreement between spectralis and Cirrus. The x-axis shows the mean RNFL thickness and the y-axis shows the difference in measurements between Spectralis global RNFL and Cirrus RNFL thickness. The solid line illustrates the mean difference (mean bias = –4.78 µm) and the dashed lines illustrate the limits of agreement. The 95% limit of agreement for the two devices was between –37.9 and 28.3 µm. There is some heteroskedasticity at values > 150 µm (more heterogeneity of variance the thicker the RNFL), but also fewer data points for moderate-severe papilloedema. **B** Bland-Altman plot of maximal height agreement between Spectralis and Cirrus. The x-axis shows the mean maximal height and the y-axis shows the difference in measurements between Spectralis and Cirrus maximal height. The solid line illustrates the mean difference (mean bias = 57.4 µm) and the dashed lines illustrates the limits of agreement. The 95% limit of agreement for the two devices was between –37.6 and 152.3 µm. Scatter analysis by inspection shows good random scatter of data points, indicating consistent differences across measurement magnitudes but larger absolute differences between devices. **C** Scatter plot of Spectralis GCL volume vs Cirrus GCC thickness. The x-axis shows the Spectralis GCL total volume measurements and the y-axis shows the Cirrus GCL-IPL thickness measurements. There is a strong positive linear correlation (r^2^ = 0.8307). Figures created in https://BioRender.com Supplementary table 1: APOSTEL 2.0 Reporting Checklist. Abbreviations: APOSTEL Advised Protocol for OCT Study Terminology and Elements, IIH idiopathic intracranial hypertension, GCC ganglion cell complex, GCL ganglion cell layer, NA not applicable, RNFL retinal nerve fibre layer, OCT optical coherence tomography.

On inspection, the Bland-Altman plot suggested good agreement of RNFL thickness between the devices (Fig. 2A) when the RNFL thickness was less than 150 µm, although skewed in favour of thicker measurements on the Cirrus platform. In contrast, at higher mean RNFL thicknesses, there was a clear effect of RNFL thickness on the magnitude of discrepancy, with measurements between the two devices diverging in favour of higher measurements on Cirrus.

Bland-Altman plot for maximum disc height measurements (Fig. 2B) was skewed in favour of higher measurements on the Spectralis platform, but the magnitude of difference did not vary with increasing height.

Spectralis RNFL and Cirrus RNFL thickness showed excellent agreement (ICC = 0.922; 95% CI 0.892–0.944; Table 3 and Fig. 2A). Spectralis maximum height and Cirrus maximum height showed good agreement (ICC = 0.882; 95% CI 0.218–0.962; Table 3 and Fig. 2B). Spectralis GCL volume and Cirrus GC + IPL thickness displayed very strong positive correlation (Pearson’s r coefficient correlation = 0.911; 95% CI 0.883–0.933; Table 3 and Fig. 2C).

**Table 3:** Intraclass Correlation Coefficient and Pearson Correlation for Spectralis global RNFL and Cirrus RNFL thickness, Spectralis maximal height and Cirrus maximal height and Spectralis GCL volume and Cirrus GC-IPL thickness.

	Intraclass Correlation Coefficient^a^ Single measures (95% CI)^b^	Significance (F test with True Value 0)	Intraclass Correlation Coefficient^a^Average measures (95% CI)^c^	Significance (F test with True Value 0)	Pearson Correlation (95% CI)^d^	Significance (2-tailed)
**Spectralis Global RNFL –Cirrus RNFL Thickness**	0.922 (0.892–0.944)	<0.001*	0.960 (0.943–0.971)	<0.001*	0.958 (0.944–0.968)	<0.001*
**Spectralis Maximum Height– Cirrus Maximum Height**	0.882 (0.218–0.962)	<0.001*	0.937 (0.357–0.980)	<0.001*	0.951 (0.935–0.963)	<0.001*
**Spectralis GCL volume –Cirrus GC-IPL Thickness**	NA		NA		0.911 (0.883– 0.933)	<0.001*

*CI* confidence interval, *GCL* ganglion cell layer, *GC-IPL* ganglion cell layer-inner plexiform layer, *NA* not applicable, *RNFL* retinal nerve fibre layer.

^a^Type A intraclass correlation coefficients using an absolute agreement definition.

^b^The estimator is the same whether the interaction effect is present or not.

^c^This estimate is computed assuming the interaction effect is absent, because it is not estimable otherwise.

^d^Estimation is based on Fisher’s r-to-z transformation.

* Correlation is significant at the 0.01 level.

## Discussion

Agreement was found between measurements of RNFL and maximum height recorded on the same day with Spectralis and Cirrus SD-OCT devices in people with IIH and low levels of papilloedema. RNFL measurements seemed to diverge with greater papilloedema severity (RNFL > 200 µm). GCL volume (Spectralis) correlated very strongly with the height of the GCL + IPL thickness (Cirrus). These data suggest broad applicability of research using RNFL measured with Spectralis and Cirrus platforms, possibly with limited applicability for RNFL measures in severe disease, some applicability of using maximum disc height, and platform-specific applicability for GCL and GCL + IPL measurements.

Overall, the RNFL correlated well between the two devices with excellent absolute agreement (ICC 0.922) with a 95% confidence interval ranging from good to excellent (0.892-0.944). In previous studies comparing RNFL measurements in healthy eyes, Spectralis reported thicker global RNFL measurements than Cirrus [21, 22]. There have been no prior studies evaluating eyes with papilloedema where the RNFL is abnormally elevated, and with the increasing number of randomized controlled trials in conditions such as IIH, this would be an important predetermination as to the feasibility of using different OCT devices for accurate outcome measurements [23]. On inspection of the Bland-Altman plot for RNFL thickness, there was obvious agreement between the two devices for RNFL below 150μm. With increasing papilloedema, in the relatively fewer data points of severe papilloedema, the measurements diverged. One explanation is that manual segmentation of the images was performed if the segmentation was erroneous when using the Spectralis; however, no such correction is available on the Cirrus device. Whilst these differences represent differences in research methodology, they also represent real-world differences and limitations[24]. Segmentation errors, particularly in RNFL scans of moderate to severe optic disc swelling, regardless of disease mechanism, are increasingly recognized both in clinical practice and research. Ideally, all OCT scans should be quality assessed and manual resegmentation performed if OCT software provides this option [25]. We have previously provided a video that demonstrates how to manually correct segmentation errors on Heidelberg Spectralis software [25] (https://www.youtube.com/watch?v=645A13WuPdE). Adhering to a set of OCT reporting guidelines, such as the APOSTEL 2.0 reporting recommendations, further upholds the quality of OCT use. Additionally, it may be more challenging to return accurate measurements based on signal differences in individual layers when the optic nerve is oedematous, resulting in less clear determination of the internal and external boundaries of the RNFL, which on Spectralis platforms can be addressed by examining total peripapillary retinal thickness, a measurement that can be obtained both by the inbuilt software and by manual resegmentation. The total peripapillary retinal thickness was not reported in our study because the Cirrus device does not provide a comparable outcome measure. Finally, elevation of the optic nerve head can surpass the device’s maximum scanning depth. While scan concentration is manually set by the Spectralis and automatically by Cirrus, in either device, it is likely that this is more challenging when the optic nerve cup is obliterated by swelling.

When analyzing maximum disc height, the Bland-Altman plot inferred good agreement between the devices, and there was a good ICC (0.882), but a wider confidence interval ranging from poor to excellent (0.218–0.962), implying low precision and high variability. Whilst the absolute difference between the devices of the maximum height measurement was larger than the differences found in the measurement of the RNFL, they differed similarly in proportion to the mean measure. The greater absolute differences could be due to dissimilarity in the top of the RNFL to the basement membrane boundary segmentation algorithm of the Spectralis and the manual drawing of the maximum height with the Cirrus, customized for this study. One reason to derive a simple measure of maximum height using the Cirrus was to provide a comparator measurement to the Spectralis maximum height, which is a relatively accessible technical solution.

The correlation between GCL and GCL + IPL was very strong, with a Pearson correlation coefficient of 0.911 and a confidence interval ranging from strong to very strong correlation (0.883- 0.933). The Spectralis measures GCL as a volume, and the Cirrus measures combined GCL and IPL height. There are no studies to date exploring the correlation between Spectralis GCL and Cirrus GCL + IPL. It was expected that the correlation of the ganglion cell measures would be lower than RNFL measures because the devices measure different structural dimensions to represent ganglion cell measurements; however, the correlation was very strong, despite only minimal structural variation within the population. As correlation is calculated as the ratio of between-subject to total variance and is increased by covariance, had there been a considerable proportion of optic neuropathy in this population, causing more pronounced ganglion cell loss, there would probably have been a greater signal-to-noise ratio with variation in GCL thickness exceeding natural variation between subjects and an even stronger correlation. Additionally, Fig. 2C emphasises that a proportion of people living with IIH and varying levels of papilloedema had already experienced ganglion cell loss at the time of study inclusion (GCL volume <1.00 mm^3^/GCC thickness <75 μm). Ganglion cell loss caused by moderate to severe papilloedema is associated with visual outcomes in IIH and thus is an essential outcome measure for any treatment aimed at vision-threatening papilloedema in IIH [3, 26, 27].

A key strength of this study was the direct evaluation of agreement between two widely used SD-OCT platforms in papilloedema within a rigorously phenotyped IIH cohort. Adherence to the APOSTEL 2.0 reporting recommendations ensured detailed documentation of acquisition protocols, device settings and analysis pipelines, supporting reproducibility and facilitating comparison with future studies [16]. The use of native manufacturer software enhances the clinical translatability of these findings, reflecting real-world implementation rather than research-specific post-processing.

This study has several limitations. We did not analyse quadrant-specific RNFL measures, which may demonstrate differential susceptibility to papilloedema-related segmentation error and warrant future investigation [28, 29]. With respect to maximum disc height, comparing a manually derived metric to a metric derived via automated device software could introduce bias, although this does reflect real-world application of this measure. Enhanced depth imaging (EDI) was not performed as Cirrus does not support a dedicated EDI acquisition mode. Future studies evaluating maximum disc height could use the lamina cribrosa as a consistent anatomical landmark and apply manual derivation across both platforms to enable direct methodological comparison. In this study, we assessed same-day OCT measurements at one time point only; future research would include a prospective study to longitudinally follow patients to assess inter-device agreement for change detection and assessment. Of note, other studies have already explored measurement-to-measurement differences, confirming good same-day test-retest repeatability in both Spectralis and Cirrus devices [8, 30]. With a larger dataset, it could be possible to plan a conversion equation between devices for RNFL thickness in people with IIH and optic nerve head elevation ranging across papilloedema of all severities.

Even though RNFL measurements were not interchangeable across devices, there was good inter-device agreement, which supports the idea that a patient can, if necessary, be followed by different clinicians using different devices. It is likely that the progression of papilloedema could be reasonably inferred across devices, as can any evidence of evolving optic neuropathy evidenced by the excellent GCL volume and GCC thickness correlation. This is clinically useful, as it serves as proof that the devices ‘speak the same language’.

In conclusion, this is the largest OCT imaging dataset to date comparing RNFL, maximal height and GCL volume and GCL + IPL thickness for Spectralis and Cirrus devices in people living with IIH across a range of optic nerve head elevation. While all three measured variables showed overall good agreement between devices (ranging from good to excellent with variable confidence intervals), none were directly interchangeable across devices, which carries implications for the design of future studies using structural OCT measures as outcomes. Although there was excellent agreement and very strong correlation for RNFL values using Spectralis and Cirrus, caution should be applied in using OCT RNFL measures from the respective devices interchangeably. It is recommended that monitoring of papilloedema should continue on the same device, as the more elevated the nerve is, the greater the divergence between device measures.

Supplemental material is available on Eye’s website.

## Summary

### What is known about this topic

- It is known, but not widely acknowledged, that measurements from commonly used spectral-domain OCT devices are different. This limits the direct applicability of research outcomes to clinical practice

### What this study adds

- It is likely that the progression of papilloedema could be reasonably inferred across devices, as can the evolving optic neuropathy evidenced by the excellent GCL volume and GCC thickness correlation. While all three measured variables showed overall good agreement between devices, none were directly interchangeable across devices. This carries implications for the design of future studies using structural OCT measures as outcomes

## Supplementary information

Supplemental Table 1 - APOSTEL 2.0 Reporting Checklist

## Data Availability

Professor SPM takes full responsibility for the data, the analyses and interpretation and the conduct of the research. She has full access to all the data and has the right to publish any and all data separate and apart from any sponsor. Proposals for data access should be made to the corresponding author. Reasonable, scientifically sound proposals, from appropriately qualified research groups, will provide data beginning 12 months and ending 3 years after publication of this article to researchers whose proposed use of the data is approved by the corresponding author. Requesters will need to sign a data access agreement, which will cover the terms and conditions of the release of data and will include publication requirements, authorship, acknowledgments, and obligations for the responsible use of data.
