# Supplementary material for: Optical coherence tomography imaging device comparison study in papilloedema secondary to idiopathic intracranial hypertension
Source: Eye (Lond). 2026 Sep 22;40(16):2633–40. doi: 10.1038/s41433-026-04823-4 (PMC13754846; doi:10.1038/s41433-026-04823-4)
Supplement: Supplementary file 1 [file 41433_2026_4823_MOESM1_ESM.docx]

| Item | Category | APOSTEL 2.0 Reporting Requirements |
| --- | --- | --- |
| 1 | Study protocol | 1. Number of OCT operating sites: 1 2. Number of graders/readers: 3 3. Timing of OCT relative to other assessments: same day (within minutes) 4. Inclusion criteria: patients from one neuro-ophthalmologist specialist clinic over 3 months, age >18, meeting modified 2013 Friedman criteria for IIH 5. Exclusion criteria: <18 years, not meeting modified 2013 Friedman IIH criteria 6. Reference to supplementary methods if word count limited: *see methods* |
| 2 | Acquisition device | For all OCT devices:   1. Manufacturer: Heidelberg; Carl Zeiss 2. Device model: Spectralis; Cirrus 6000 3. Hardware version: Spectralis HRA OCT; Zeiss Cirrus 6000 4. Software version: HRA Spectralis 6.12.3.0; Cirrus 11.5.25432 5. OCT technology: spectral-domain |
| 3 | Acquisition settings | 1. Pupil dilation prior to imaging: Spectralis no; Cirrus no 2. Number of operators: 2 senior ophthalmic technicians 3. Number of devices used: one Heidelberg Spectralis, one Zeiss Cirrus 6000 |
| 4 | Scanning protocol *(Please see figure 1*) | 1. Scan type (circular, volume, star, line, other): *Table 1* 2. Anatomical location (macula, optic nerve head): *Table 1* 3. Scan parameters: *Table 1* 4. Scan size and area (degrees/mm): *Table 1* Number of B-scans: *Table 1* Number of A-scans per B-scan: *Table 1* Eye tracking used (yes/no): Spectralis yes; Cirrus yes (**FastTrac)** 5. Ring scan details (diameter, centring method): *Table 1* 6. Line scan details (angle, depth resolution): *Table 1* |
| 5 | Funduscopic imaging | 1. Additional imaging modalities used (fundus photography, FAF, angiography, cSLO): NA 2. Acquisition protocol: NA 3. Device-specific features used (e.g. enhanced depth imaging): NA |
| 6 | Post acquisition data selection | 1. Image quality control criteria: >15 (Spectralis); ≥6/10 (Cirrus) 2. Number of scans excluded and reasons: 0/561 Spectralis scans excluded (21/582 scans missing: 3 patients left before Spectralis imaging was performed (18 scans), 1 patient had repeat RNFL instead of ONHV (1 scan), 1 patient had no ONHV (2 scans); 0/582 Cirrus scans excluded, 0/582 Cirrus scans missing 3. Eye selection strategy: data from both eyes used |
| 7 | Post acquisition analysis | 1. Software used for image processing and segmentation: Spectralis viewing module (software number 6.12.3.0); Cirrus viewing module (software number 11.5.25432) 2. Retinal layers segmented/analysed: GCL; GCC 3. Segmentation method (automated, semi-automated, manual): automated and manual (Heidelberg); automated (Zeiss) 4. Handling of segmentation errors or bias: manual resegmentation (Heidelberg) 5. Measurement grid used (size, shape, regions): *Table 1* 6. Pixel-to-millimeter conversion if applicable: NA |
| 8 | Nomenclature and abbreviations | 1. Anatomical structures analysed: RFNL thickness; maximal height, GCC thickness, GCL volume 2. Units of measurement (thickness, volume): μm (RNFL, maximal height, GCC), mm^3^ (GCL) 3. Number of eyes with additional retinal pathology: 0 4. Description and quantification methodology for additional pathology: NA |
| 9 | Statistical approach | 1. Statistical models used for OCT analyses: Intraclass correlation coefficient, Pearson correlation 2. Unit of analysis (eye-based vs patient-based): eye-based 3. Handling of inter-eye correlation if applicable: eyes treated as unlinked |
